# Supplementary material for: Domain adaptable language modeling of chemical compounds identifies potent pathoblockers for Pseudomonas aeruginosa
Source: Commun Chem. 2025 Apr 11;8:114. doi: 10.1038/s42004-025-01484-4 (PMC11992043; doi:10.1038/s42004-025-01484-4)
Supplement: Supplementary file 2 — Supplementary Information [file 42004_2025_1484_MOESM2_ESM.pdf]

Supplementary Information

Domain adaptable language modeling of chemical compounds identifies potent pathoblockers for *Pseudomonas aeruginosa.*

Georgios Kallergis, Ehsannedin Asgari, Martin Empting, Anna K. H. Hirsch, Frank Klawonn and Alice C. McHardy*.

Algorithm 1 Pseudocode for SMILES enumeration algorithm

```
nm ← []
number_augmentations ← 100
counter ← 0
for all smiles ∈ smiles_list do
    for counter ← 0 to number_augmentations − 1 do
        atom_numbers ← smiles.get_number_atoms()
        m ← shuffle(atom_numbers)
        nm.extend(renumberAtoms(m, atom_numbers))
    end for
end for
```

Supplementary Table 1 Values range that was utilized for the hyperparameter optimization.

Parameter	Values range
Augmentation size	0,5,10,15,20,25, 40, 60, 80, 100
Number of hidden layers	4, 8, 12
Number of attention heads	8, 12, 16
Embeddings	Pooling<br>Last layer - mean of tokens<br>Last layer -first token<br>Sum of hidden layers - mean of tokens<br>Sum of hidden layers - first token<br>Mean of hidden layers - mean of tokens<br>Mean of hidden layers - first token

Supplementary Table 2 Training time with regard to the augmentation size for the domain adaptation training stage.

Augmentation size	Training time(s)
0	668
20	11083
40	26256
60	34731
80	44800
100	55594

Our findings suggest that the training time is linear to the augmentation size. The required time is quite low and is not discouraging from using more representations.

Supplementary Table 3 Selected hyperparameters for our models.

Model	Augmentation number	Embeddings type	Number of hidden layers	Number of attention heads
vanilla	-	Pooling	12	12
domain-adapted	80/100	Pooling	12	12
BBBP	80	Last layer -first token	8	12
BACE	100	Sum of hidden layers - mean of tokens	8	12
ClinTox	80	Last layer -first token	12	16
PA	100	Sum of hidden layers - mean of tokens	4	12

In the vanilla model, default hyperparameters from HuggingFace were utilized. The domain-adapted model shares the same values with vanilla, except for the augmentation number, in which the optimal value for each dataset was used. We identified the best hyperparameters for benchmark datasets (BACE, BBBP, ClinTox) through optimization on the validation dataset. Regarding the model for *Pseudomonas aeruginosa* (PA), the lack of validation dataset did not allow us to follow a similar procedure. We selected the best hyperparameters according to the values derived from the successful configurations identified on benchmark datasets, except that we used fewer layers, because of the small training set size. In addition, we also investigated another setting for the embeddings type (embeddings of the first token of the last layer), a larger number of hidden layers (12 instead of 4) and a lower augmentation number (80). Results are shown for the model with the best performance, selecting the best of these models.

Supplementary Table 4 Table of publications and patents detailing each structural class in the experimental data.

Structural class	Publication	Patent
A	Schütz *et al*[1], Abdelsamie *et al*[2]	WO2020007938A1 [3]<br>WO2021136805A1 [4]<br>WO2021136803A1 [5]
B		WO2020007938A1 [3]<br>WO2021136803A1 [5]
C	Schütz *et al* [6], Zender *et al*[7]	
D		
E	Hamed *et al* [8], Zender *et al*[7]	WO2020007938A1 [3]<br>WO2021136805A1 [4]<br>WO2021136803A1[5]

Supplementary Table 5 Participation of each structural class in the 5-branch setting and its percentage of highly potent compounds.

Hierarchical folds	A	B	C	D	E	Highly potent compounds	Number of compounds
1	36	29	0	0	13	88%	78
2	40	15	1	1	6	67%	63
3	20	4	2	1	22	40%	49
4	0	0	11	0	8	21%	19
5	10	0	0	0	0	50%	10

Supplementary Table 6 Performance comparison of property prediction models over the test set of a 5-fold cross-validation setting over the experimental dataset. The macro-averaged score is reported for each metric.

Model	F1	AUC	Precision	Recall	Accuracy
MolFormer	0.45	0.49	0.47	0.47	0.73
MolBERT	0.5	0.5	0.55	0.6	0.71
ChemBERTa	0.33	0.5	0.25	0.5	0.5
MPNN	0.60	0.59	0.66	0.59	0.79
GAT	0.56	0.58	0.64	0.58	0.71
GCNN	0.57	0.57	0.58	0.57	0.65
ChemLM	0.90	0.90	0.90	0.90	0.90

The median metric value of each model is demonstrated.

Supplementary Table 7 Predictive performance of ChemLM and state-of-the-art models on the positive class (highly potent pathoblockers).

Hierarchical Folds	ChemLM	MPNN	GAT	GCNN	MolBERT	MolFormer	ChemBERTa
1	0.92	0.94	0.92	0.91	0.87	0.45	0.47
2	0.83	0.8	0.82	0.77	0.78	0.45	0.4
3	0.90	0.72	0.62	0.63	0.70	0.73	0.29
4	0.89	0.33	0.00	0.75	0.00	0.42	0.17
5	0.89	0.75	0.73	0.00	0.60	0.67	0.33

The F1-score is reported as evaluation metric in this this table.

Supplementary Table 8 Description of the evaluation datasets.

Datasets	Number of compounds	Percentage of positive class
BACE	1513	45.7%
BBBP	2039	76.5%
ClinTox	1478	7.6%

Supplementary Table 9 Comparison of ChemLM on BBBP dataset with its simpler versions, and state-of-the-art models in more evaluation metrics. The macro-averaged score is reported for each metric.

Model	F1	AUC	Precision	Recall	Accuracy
MolFormer	0.92	0.71	0.89	0.71	0.86
MolBERT	0.89	0.89	0.9	0.89	0.93
ChemBERTa	0.87	0.89	0.85	0.89	0.9
MPNN	0.78	0.79	0.78	0.79	0.84
GAT	0.75	0.71	0.85	0.71	0.85
GCNN	0.7	0.66	0.82	0.66	0.83
ChemLM vanilla	0.69	0.67	0.72	0.67	0.8
ChemLM domain-adapted	0.82	0.81	0.837	0.81	0.87
ChemLM domain-adapted & optimized	0.88	0.89	0.87	0.88	0.91

Supplementary Table 10 Comparison of ChemLM on BACE dataset with its simpler versions, and state-of-the-art models in more evaluation metrics. The macro-averaged score is reported for each metric.

Model	F1	AUC	Precision	Recall	Accuracy
MolFormer	0.9	0.91	0.91	0.91	0.91
MolBERT	0.81	0.81	0.81	0.81	0.82
ChemBERTa	0.69	0.69	0.69	0.69	0.69
MPNN	0.73	0.73	0.73	0.73	0.73
GAT	0.67	0.70	0.77	0.70	0.68
GCNN	0.69	0.69	0.73	0.69	0.71
ChemLM vanilla	0.51	0.55	0.60	0.55	0.58
ChemLM domain-adapted	0.66	0.66	0.67	0.66	0.67
ChemLM domain-adapted & optimized	0.80	0.80	0.80	0.80	0.81

Supplementary Table 11 Comparison of ChemLM on benchmark datasets with state-of-the-art models in prediction of the positive class using F1-score as evaluation metric.

Model	ClinTox	BACE	BBBP
MolFormer	0.55	0.90	0.92
MolBERT	0.38	0.8	0.96
ChemBERTa	0.81	0.67	0.93
MPNN	0.31	0.72	0.90
GAT	0.22	0.73	0.91
GCNN	0.35	0.61	0.90
ChemLM domain-adapted & optimized	0.84	0.79	0.94

Supplementary Table 12 Results of the t-test used to compute p-value for each molecular property.

Molecular Property	t-statistic	df	CI	p -value
Molecular Weight	17.23	99	5.49, inf	7.48e-32
QED	36.95	99	0.01, inf	5.05e-60
Polar surface area	8.53	99	1.33, inf	8.74e-14

P-values were computed using a one-tailed t-test to determine if the mean of the ChemLM ratio distribution was significantly greater than that obtained from random shuffling of the molecular property labels. The term ”df” represents degrees of freedom, while ”CI” refers to the confidence interval.

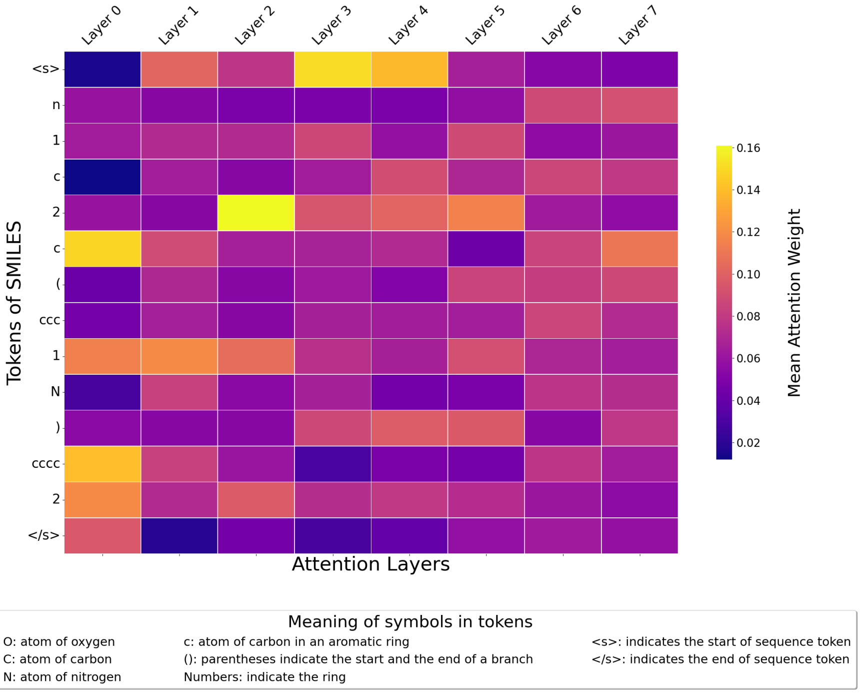

Supplementary Fig. 1 Heatmap of the attention distribution in the tokens of a SMILES sequence. It depicts the sum of attention a token receives from all attention heads in each layer of the model.

References

[1] Schütz, C., Ho, D.K., Hamed, M.M., Abdelsamie, A.S., Röhrig, T., Herr, C., Kany, A.M., Rox, K., Schmelz, S., Siebenbürger, L., Wirth, M., Börger, C., Yahiaoui, S., Bals, R., Scrima, A., Blankenfeldt, W., Horstmann, J.C., Christmann, R., Murgia, X., Koch, M., Berwanger, A., Loretz, B., Hirsch, A.K.H., Hartmann, R.W., Lehr, C.M., Empting, M.: A New PqsR Inverse Agonist Potentiates Tobramycin Efficacy to Eradicate Pseudomonas aeruginosa Biofilms. Advanced Science **8**(12) (2021). https://doi.org/10.1002/ADVS.202004369

[2] Abdelsamie, A.S., Hamed, M.M., Schütz, C., Röhrig, T., Kany, A.M., Schmelz, S., Blankenfeldt, W., Hirsch, A.K.H., Hartmann, R.W., Empting, M.: Discovery and optimization of thiazole-based quorum sensing inhibitors as potent blockers of pseudomonas aeruginosa pathogenicity. Eur. J. Med. Chem. **276**, 116685 (2024). https://doi.org/10.1016/j.ejmech.2024.116685

[3] PqsR Inverse Agonists 2018 Ref. No: WO2020007938A1 (EP18181475)

[4] Novel PqsR Inverse Agonists 2020 Ref. No: WO2021136805A1 (EP20150119).

[5] New PqsR Inverse Agonist 2020 (EP20150104).

[6] Schütz, C., Hodzic, A., Hamed, M., Abdelsamie, A.S., Kany, A.M., Bauer, M., Röhrig, T., Schmelz, S., Scrima, A., Blankenfeldt, W., Empting, M.: Divergent synthesis and biological evaluation of 2-(trifluoromethyl)pyridines as virulence-attenuating inverse agonists targeting PqsR. European journal of medicinal chemistry **226** (2021). https://doi.org/10.1016/J.EJMECH.2021.113797

[7] Zender, M., Witzgall, F., Kiefer, A., Kirsch, B., Maurer, C.K., Kany, A.M., Xu, N., Schmelz, S., Börger, C., Blankenfeldt, W., Empting, M.: Flexible Fragment Growing Boosts Potency of Quorum-Sensing Inhibitors against Pseudomonas aeruginosa Virulence. Chemmedchem **15**(2), 188 (2020). https://doi.org/10.1002/CMDC.201900621

[8] Hamed, M.M., Abdelsamie, A.S., Rox, K., Schütz, C., Kany, A.M., Röhrig, T., Schmelz, S., Blankenfeldt, W., Arce-Rodriguez, A., Borrero-de Acuña, J.M., Jahn, D., Rademacher, J., Ringshausen, F.C., Cramer, N., Tümmler, B., Hirsch, A.K.H., Hartmann, R.W., Empting, M.: Towards Translation of PqsR Inverse Agonists: From In Vitro Efficacy Optimization to In Vivo Proof-of-Principle. Advanced Science, 2204443 (2023). https://doi.org/10.1002/ADVS.202204443
